# No Short-Term Effects of Acromioclavicular Joint Augmentation in Acute Acromioclavicular Joint Stabilization Surgery: A Randomized Controlled Clinical Trial on 70 Patients

**DOI:** 10.3390/jcm14093161

**Published:** 2025-05-02

**Authors:** Miha Ambrožič, Matej Cimerman, Kristjan Omahen, Martina Jaklič, Veronika Kralj-Iglič, Ladislav Kovačič

**Affiliations:** 1 Centre for Clinical Research, University Medical Centre Ljubljana, 1000 Ljubljana, Slovenia; 2 Medical Faculty, University of Ljubljana, 1000 Ljubljana, Slovenia; 3 General Hospital Celje, 3000 Celje, Slovenia; 4 Faculty of Health Sciences, University of Ljubljana, 1000 Ljubljana, Slovenia; 5 Arbor Mea Medical, 1000 Ljubljana, Slovenia; ladislav.kovacic@gmail.com

**Keywords:** acromioclavicular joint dislocation, horizontal acromioclavicular instability, acromioclavicular augmentation, overlapping length, overlapping area, coracoclavicular distance, arthroscopic assisted surgery

## Abstract

**Background**: Optimal treatment for high-grade acromioclavicular (AC) joint dislocations is still not unanimous. Improving horizontal AC stability has been emphasized in recent years. Biomechanical studies and computer simulations have demonstrated that adequate horizontal stability could be restored with an additional AC fixation. We aim to prospectively investigate if AC augmentation leads to better clinical and radiological results. **Methods**: A total of 70 patients with a mean (± SD) age of 42 ± 11 years with acute AC joint dislocation Rockwood type IIIb and V were prospectively randomized into two equal groups. All patients underwent arthroscopically assisted stabilization using a double coracoclavicular (CC) suspensory system. Group N (No-augmentation group) had no additional fixation across the AC joint, while group T (tape-augmentation group) had additional fixation with tape. Patients were evaluated at 3, 6, and 12 months postoperatively. Primary clinical outcome measures included the Constant–Murley score and the Specific AC Score (SACS). Secondary outcome measures included the Subjective Shoulder Value (SSV), the Simple Shoulder Test (SST), the Disabilities of the Arm, Shoulder, and Hand (DASH) outcome measure, and the AC Joint Instability (ACJI) Score. Horizontal stability was radiologically measured with overlapping length (OL) and overlapping area (OA). Vertical alignment was measured with the CC distance. All radiological measurements were compared to the uninjured side and expressed in percentages as relative values. **Results:** There were no significant differences found between groups regarding the Constant score (*p* = 0.664), SACS (*p* = 0.518), or any other outcome measure at the one-year follow-up. Pain level (*p* = 0.635) and strength (*p* = 0.217) at the one-year mark also showed no significant differences. Clinical drawer testing for residual horizontal instability was non-significant (*p* = 0.061), but showed a tendency for a more stable AC joint in group T. The CC distance was smaller in group T at 6 and 12 months (*p* = 0.047 and *p* = 0.046, respectively). A two-way mixed factorial ANOVA test showed significantly lower CC differences for group T (*p* = 0.032); however, the gradual increase in CC distance was similar for both groups over time (*p* = 0.869). No significant differences were found in OL (*p* = 0.619) or OA (*p* = 0.236). **Conclusions:** The results of our study show that both CC stabilization with the double suspensory system alone and with additional AC fixation are effective surgical treatment options for acute AC joint dislocations, without any important clinical differences. CC distance similarly increased over one year in both groups but was better retained in the AC-augmented group, which showed a tendency toward a more stable fixation.

## 1. Introduction

Acromioclavicular (AC) joint dislocations are frequent injuries among the sport-active population, mostly males [1,2,3,4,5]. In higher grades, a preferred treatment is surgical stabilization [6]. Classic fixation methods, such as Bosworth screw, K-wire fixation, and hook plates have been largely replaced with more anatomic coracoclavicular (CC) reconstructions (ACCR) in the hope of decreasing complication rates and improving patient outcomes [6,7,8,9]. One major advantage is no need for hardware removal in a second surgery, as well as less risk for neurovascular damage, less blood loss and potentially shorter operative time in minimally invasive arthroscopic techniques [10,11].

Controversy regarding Rockwood type III remains, although surgical stabilization of horizontally unstable subtype IIIb is usually preferable [7,12]. The optimal surgical technique for treating high-grade AC joint dislocations still vexes shoulder surgeons to this day. Coracoclavicular (CC) suspensory system stabilization is now a well-established and frequently performed procedure [1,13,14,15,16,17,18,19]. The double suspensory system technique, as an anatomy-mimicking reconstruction, has been proven to be superior to a single fixation, with good horizontal stability control [20]. Even so, it was reported that persisting dynamic posterior translation (DPT) appears in up to half of patients with double suspension stabilization and is associated with inferior radiological and clinical results, with more pain and worse patient outcome measures [16,18,21,22]. Focus has thus been gradually redirected into diagnosing and improving horizontal stability. The role of AC ligaments in resisting posterior displacement and axial rotation has first been described by Fukuda in 1986 [23]. Since then, the integrity of the AC capsule has been additionally emphasized [24,25,26], and new radiological measurement methods have been developed to assess horizontal instability [27,28,29,30]. Biomechanical [31,32] and digital finite element analysis studies [33] have shown superior horizontal stability with additional AC cerclage, as well as important load-sharing protective effect towards CC suspensory system. Many surgeons have since decided to utilize it. It has been found that development of complete DPT was 4.8 times more likely in patients who underwent isolated CC stabilization than in patients with additional AC stabilization [21], and a lower incidence of DPT compared to other studies was observed in a prospective analysis of patients with combined CC and AC stabilization [34].

This study aims to clarify if adding AC fixation in AC joint stabilization surgery is significantly better compared to CC fixation alone. We hypothesized that patients who had AC joint augmentation would have better clinical and radiological results.

## 2. Materials and Methods

**Figure 1:**
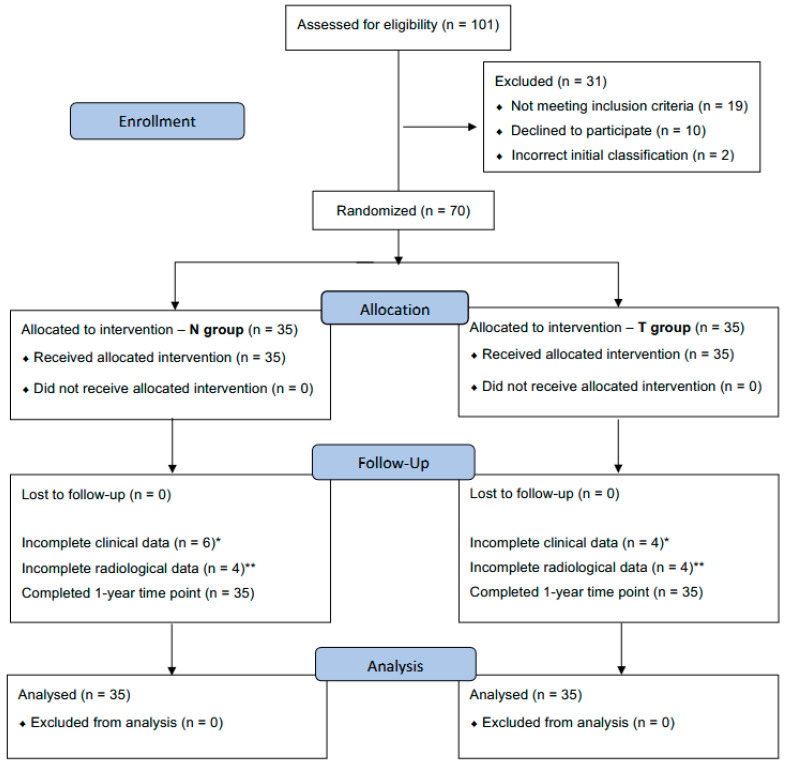
CONSORT flow diagram of assignment to group T (additional tape augmentation) and group N (no additional augmentation). CONSORT, Consolidated Standards of Reporting Trials. * Patients have not fulfilled questionnaires or data in physical form have been lost prior to transforming them into a digital spreadsheet. ** Alexander view X-rays had not been taken at the 3- or 6-month time points, or substandard quality projections were obtained and were not applicable for measurements.

This study is a two-center, double-blinded, randomized controlled clinical trial. It was approved by the National ethics committee in Slovenia on 29 March 2021 under reference number 0120-577/2020/4 and performed in accordance with the World Medical Association Declaration of Helsinki (JBJS 79-A: 1089-98, 1997). Written informed consent was obtained from all participants prior to inclusion. The study was reported beforehand to ClinicalTrials.org under reference number NCT05143853 (registration date: 29 April 2021). Study was executed in accordance with CONSORT guidelines (Figure 1).

### 2.1. Patient Allocation, Study Design, and Eligibility Criteria

**Figure 2:**
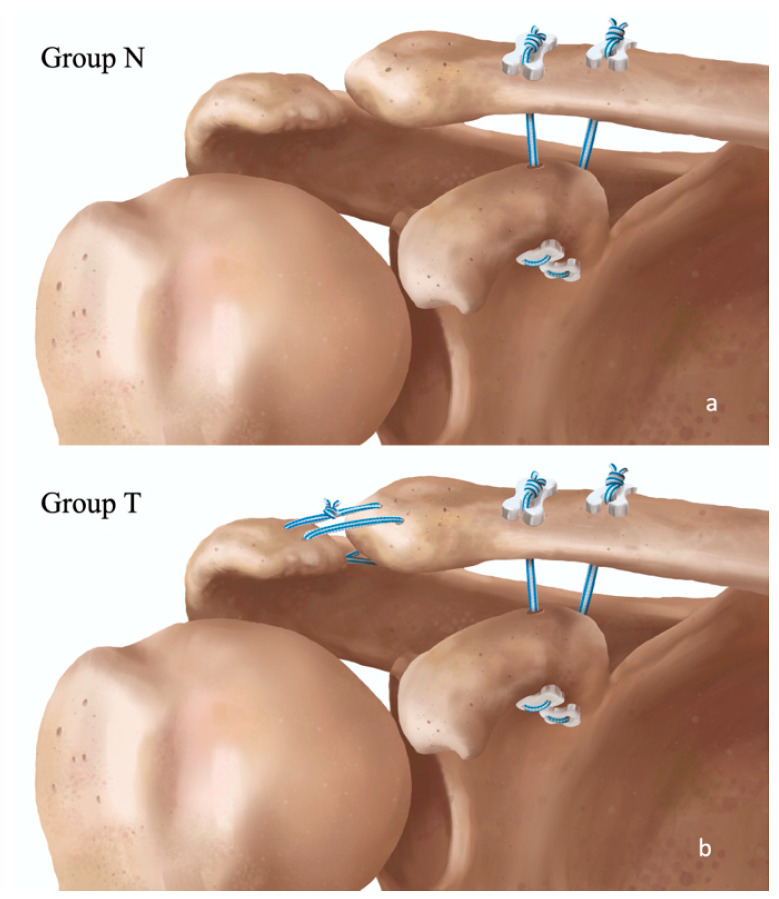
3D drawing of a double suspension CC stabilization without AC augmentation in group N (**a**) and with AC augmentation in group T (**b**), displayed as crossing tape limbs under the AC joint and parallel on the superior side, with the knot tied posteriorly on top. Drawings by Luka Hodnik.

From April 2021 through February 2023, 70 consecutive patients with acute AC joint dislocation were treated surgically at the University Medical Centre Ljubljana and the General Hospital Celje. Rockwood type IIIb and above were considered for inclusion. The final differentiation between IIIa and IIIb types was determined preoperatively with the cross-body adduction test by one of the treating surgeons [12] and with bilateral modified Alexander X-ray projections [29]. Inclusion and exclusion criteria are presented in Table 1. A double button-tape suspensory system was used for clavicle fixation to the coracoid in all patients. One system incorporated two Dog Bone^TM^ buttons, one FiberTape^®^, and one TigerTape^TM^ (Arthrex, Inc., Naples, FL, USA). Two groups of patients, equal in quantity (35 vs. 35), were formed based on additional fixation of the clavicle to the acromion (Figure 2) Group N included patients without AC augmentation, whereas group T comprised patients with AC tape augmentation (FiberTape^®^; Arthrex, Inc., Naples, FL, USA) (Figure 2). An online program (www.randomizer.org, accessed on 29 April 2021) was used to generate a randomized list for allocating patients to the two groups. Patients were blinded to the treatment type throughout the follow-up, while surgeons did not open concealed envelops with randomization numbers until entering the operating room. Follow-up time points were set at 3, 6, and 12 months postoperatively. Examination, functional tests, and radiological measurements were performed by two independent reviewers, who were also blinded to the treatment type.

### 2.2. Surgical Technique

**Figure 3:**
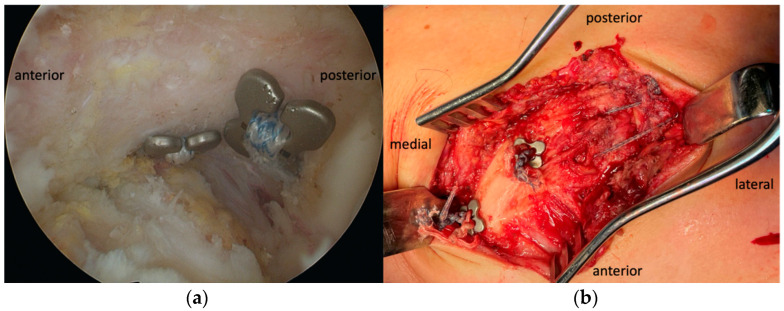
Dog Bone buttons with tapes on the undersurface of the coracoid base—arthroscopic view (**a**), Dog Bone buttons with knotted tapes on the superior side of the clavicle and AC joint augmentation with parallel tape strands on the superior side, with a knot positioned posteriorly (**b**).

All surgeries were performed by one of two senior shoulder surgeons (M.A. and K.O.). Open reduction of the AC joint was performed with arthroscopically assisted drilling of bone tunnels in place of conoid and trapezoid ligaments (4.5 and 2.5 cm from the lateral clavicle end) and into the base of the coracoid. Two suspensory systems were shuttled in a retrograde direction and knotted over two additional buttons on the superior side of the clavicle (Figure 2 and Figure 3). In group T, a non-absorbable tape was also placed through bone tunnels on each side of the AC joint and tied firmly (Figure 2 and Figure 3b). Because of the acute setting, no additional biological material was incorporated in the construct [35]. AC capsule repair was incorporated in the muscle–fascia suture closure in all patients in both groups. A detailed description of the surgical technique is available in the Supplementary Materials.

### 2.3. Postoperative Rehabilitation

A bilateral, non-weighted Zanca view X-ray (AP projection with 15° cephalad tilt) was taken after surgery to confirm proper joint reduction and implant position. Patients were instructed to avoid weight-bearing with the arm in a shoulder brace for 4 weeks after surgery. Passive range of motion (ROM) was permitted immediately with active-assisted exercises commenced after 2 weeks. Active ROM was permitted after 6 weeks, and muscle-strengthening exercises after 3 months. Encouragement for normalization of physical activities was recommended after 6 months.

### 2.4. Clinical Assessment

The first clinical inspection was made 1 month after discharge. During the visit, patients were inspected for primary wound healing; any signs of local infection were described and treated accordingly. After every subsequent control exam (3, 6, and 12 months after surgery), ROM was evaluated, and strength values were gathered as part of functional outcome measures. Scapular dyskinesis tests were not routinely performed.

Primary clinical outcome measures included the Constant–Murley score (Constant score) and the Specific Acromioclavicular Score (SACS). Secondary clinical outcomes included the Disabilities of the Arm, Shoulder, and Hand (DASH) questionnaire, the Subjective Shoulder Value (SSV), the Simple Shoulder Test (SST), and the Acromioclavicular Joint Instability (ACJI) score. SACS [36] and ACJI [18] questionnaires were specifically designed to assess AC joint pathology, while the others are general shoulder and upper extremity scores. Strength was measured with a digital dynamometer (Meilen Life, Shenzhen, China) with the arm in a 90° abduction position in the scapular plane. Bilateral strength measurements were performed with five repetitions. Residual horizontal instability was clinically tested by the examiner with a posterior drawer test for the lateral clavicle at the final follow-up. The outer borders of the lateral clavicle and acromion were outlined with a skin marker, and the center of the AC joint location was identified and marked. The lateral clavicle was grasped with the thumb and index finger and moved posteriorly relative to the acromion. Instability was judged as none if the clavicle failed to move relative to the center line of joint, as partial if it moved less than one clavicle’s width, and as complete if it moved more than one clavicle’s width. Pain level was measured on a self-evaluation scale from 0 to 15, where 15 represented the worst pain. Strength, expressed as a percentage compared to the healthy side, was analyzed separately at the final follow-up. Pain level and strength data were extracted from the Constant score and analyzed separately.

### 2.5. Radiological Assessment

**Figure 4:**
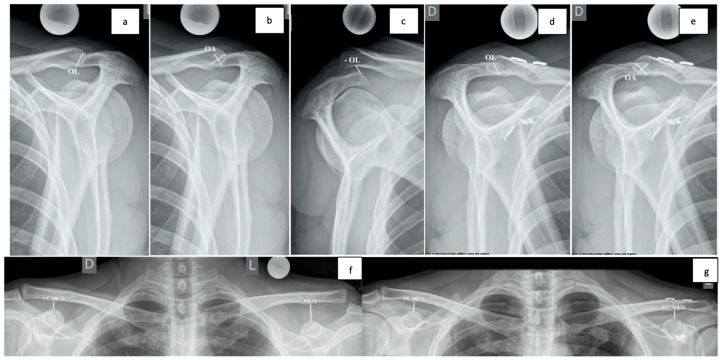
Modified Alexander view with the arm in a cross-body adduction position (**a**–**e**), bilateral Zanca projection (**f**,**g**). Unaffected shoulder with a demonstration of OL (**a**) and OA measurements (**b**), complete horizontal translation with overriding clavicle, with a demonstration of a negative OL measurement (**c**), AC joint after reduction and fixation with a double suspensory system and OL (**d**) and OA measurements (**e**), complete AC joint dislocation on the left side (Rockwood type V) with CC measurements (**f**), AC joint after reduction and fixation with intended overreduction on the left side and CC measurements (**g**). OL—overlapping length; OA—overlapping area; CC—coracoclavicular distance.

A bilateral, non-weighted Zanca view and modified Alexander views on both sides were obtained at each follow-up visit. The primary radiological objective was to quantify the DPT on Alexander views by measuring Overlapping Length (OL) in mm and Overlapping Area (OA) in mm^2^, as described by Minkus et al. (Figure 4). These measurements were then analyzed as relative numbers—every measurement was compared to the contralateral side on a bilateral X-ray and expressed as a percentage. Relative measurements are more representative because of differences in patient anatomy and minor mistakes in acquiring correct X-ray projections. The secondary radiological objective was to assess vertical alignment by measuring the CC distance on the Zanca view. CC distance was measured as a line from the highest part of the coracoid to the inferior cortex of the clavicle, parallel to the spine. Measurements were, again, side-comparative and expressed as percentages. Every measurement was calibrated with a 25 mm metallic sphere.

### 2.6. General Assesment

Demographic data, mechanism of injury, time from injury to surgery, duration of surgery, incision length, incidence of comorbidities and smoking were analyzed and compared between groups. Heavy smoking was defined as more than 20 cigarettes a day. Patient satisfaction with the result of treatment was self-assessed at the final follow-up on a scale from 1 to 10 (10 meaning very satisfied with the result). Surgical complications, such as wound dehiscence, infection, iatrogenic fractures, implant irritation, or loss of stability with the need for revision surgery were reported. Tunnel widening, heterotopic ossifications in the CC area, and post-traumatic AC arthrosis were not considered complications but common and expected side effects of the surgery. Major loss of CC distance, with more than 100% loss compared to the uninjured side, was analyzed separately and incorporated into the complication rate.

### 2.7. Statistical Analysis

Statistical analysis was performed using SPSS v25.0 (IBM, SPSS Inc., Chicago, IL, USA). The Shapiro–Wilk test was used to assess the normality of data distribution. The Pearson’s correlation coefficient was applied to assess the relationship between continuous variables, and Spearman’s rho correlation coefficient was used to detect a relationship between categorical data. Continuous variables were reported as the mean ± standard deviation (SD) if normally distributed, or as the median with the interquartile range (IQR: Q1–Q3) if not normally distributed. Categorical variables were summarized as counts (n) and percentages (%), including clinical outcome scores such as Constant, DASH, ACJI, SACS, SST, and SSV, as well as other parameters such as age, patient satisfaction, incision length, and surgery time. The level of significance was defined as *p* < 0.05. An a priori power analysis was performed based on the minimal clinically important difference (MCID) in the Constant score. Assuming a difference of 10 points, a standard deviation of 11, a power of 80%, and α = 0.05, a sample size of 30 per group was estimated to be sufficient. While the study was adequately powered for primary outcomes, it may have been underpowered for radiological analyses, a shortcoming that is acknowledged in the limitations section [29,37]. Data inspection revealed that most outcome variables, including pain, Constant, and SACS, were not normally distributed. Therefore, nonparametric Mann–Whitney U tests were conducted for between-group comparisons at each follow-up time point (3, 6, and 12 months). This approach avoids the assumptions of parametric methods and provides a robust comparison of clinical outcomes between groups. In addition, for selected outcomes, a two-way mixed factorial ANOVA was conducted to assess the effects of “follow-up time” (within time period: before surgery, 3, 6, and 12 months after surgery) and “groups” (between group N and group T), as well as interaction effects of “follow-up time × groups”. This analysis was used to explore overall trends and patterns over time and to complement the between-group comparisons with a longitudinal perspective.

## 3. Results

Out of 101 patients assessed for eligibility, 70 patients were randomized into two equal groups. The mean age of both groups was 42 years, and only one patient was female (in group N). Out of 70 patients, 21 (30%) patients were classified as Rockwood type IIIb, and 49 (70%) patients as type V AC dislocation. No type IV or type VI injuries were encountered during the inclusion period. The right side was affected in 39 (55.7%) cases altogether. Direct fall on the affected shoulder was described as the main mechanism of injury, with cycling (20%) and skiing (27.2%) as the most common sports. No significant differences between groups in the mechanism of injury were observed (Chi-test, *p* = 0.814). All patients were in good health, with some of them having previous medication for comorbidities. Two patients in group T had arterial hypertension (AH), and six patients in group N had other comorbidities (AH: two patients, benign prostatic hyperplasia and AH: one patient, hyperlipidaemia: one patient, hypothyroidism: one patient, and past myocardial infarction: one patient). One patient in each group declared themselves as a heavy smoker. The mean time from injury to surgery was 11.4 days (range from 2 to 21 days). There were no statistically significant differences between groups. Demographic data are presented in Table 2.

There were no differences between groups regarding incision length (*p* = 0.422) or surgical time (*p* = 0.121) based on the Mann–Whitney U test. The complete results are reported in Table S1.

### 3.1. Functional Results

Clinical outcomes were evaluated at 3, 6, and 12 months postoperatively. There were no statistically significant differences found in Constant (*p* = 0.664) or SACS (*p* = 518) (Table 3) or in any of the secondary outcomes: SSV (*p* = 0.607), SST (*p* = 0.789), DASH (*p* = 0.443), ACJI (*p* = 0.346), and strength (*p* = 0.257). The complete results can be found in Table S2.

**Figure 5:**
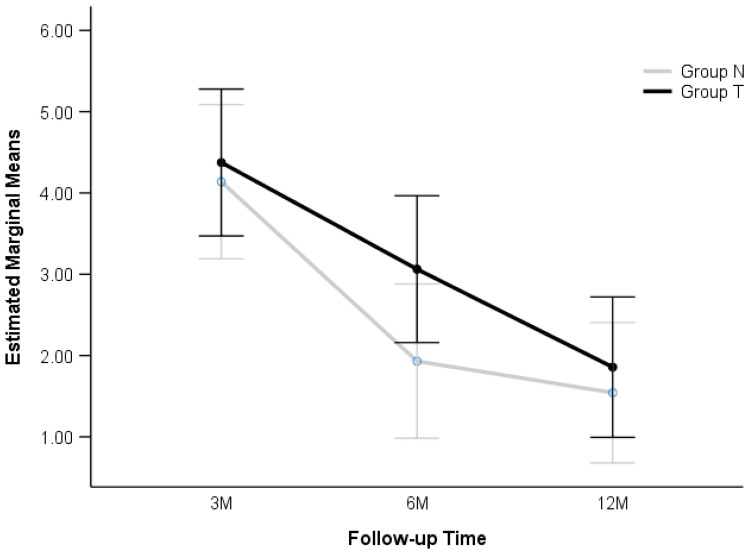
Estimated marginal means of pain level decrease over 3, 6, and 12 months for groups N and T with 95% confidence intervals (CI). M—months.

Despite overall similar outcomes produced with PROMs, there were some minor but noted clinical differences between groups. A lower level of pain was recorded in group N at 6 months (U = 531, *p* = 0.046; Mann-Whitney U Test). No statistically significant differences were observed between the groups at 3 months (*p* = 0.298) or 12 months (*p* = 0.236). Factorial ANOVA was additionally used to assess changes over ”follow-up time” and interaction effects. It showed a substantial decrease in pain level over time (F (2186) = 16.335, *p* < 0.001). However, the overall level of pain was not significantly different between groups when considering all postoperative records (F (1186) = 2.238, *p* = 0.136). The pattern of pain reduction was similar over time for both groups (F (2186), *p* = 0.568), suggesting that the estimated marginal means in group T were slightly higher (3.098) than those of group N (2.537), but this difference was not statistically significant (Figure 5).

Subjectively, patients in both groups were similarly satisfied with the final result of treatment (*p* = 0.508, Mann–Whitney U test).

There was no significant difference observed in residual horizontal instability at the 1-year time point with respect to the clinical drawer testing. However, due to a marginal *p*-value (*p* = 0.061; Chi-square test), there was a tendency for a more horizontally stable construct in group T (Table 4).

### 3.2. Radiological Results

Due to reasons explained in the *Radiological assessment* section in the Methods, we present only results from the analysis of relative numbers (expressed in percentages). The analysis of absolute numbers is available in Table S3 and Figure S1.

### 3.3. CC Measurements

**Figure 6:**
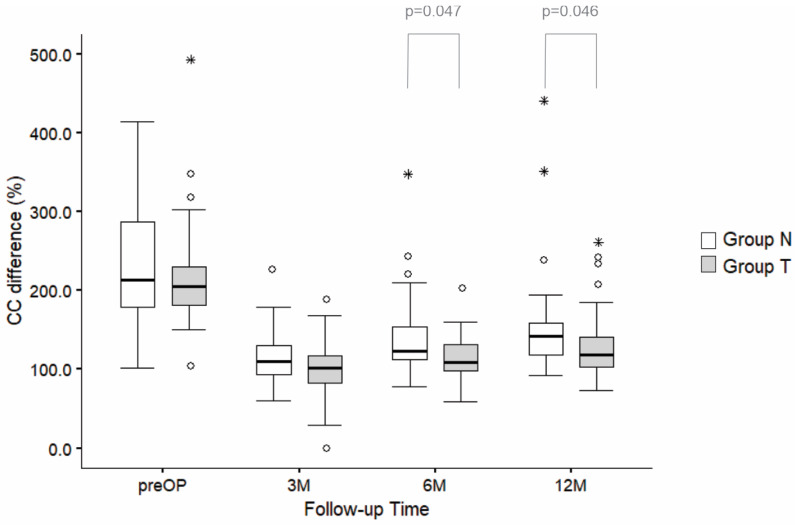
Comparison of the relative CC difference (%) between group N and group T across follow-up time points (before surgery, 3 M, 6 M, and 12 M after surgery). The boxplots represent the distribution of the CC distance in percentages for groups N and T, with horizontal lines showing the median values and whiskers representing 1.5 times the interquartile range (IQR). Statistically significant results are displayed above 6M and 12M as *p*-values. Outliers are displayed as circles (o), while asterisks (*) indicate extreme values. M—months.

Statistically significant lower values of CC differences in group T were observed at 6 months (*p* = 0.047) and 12 months after surgery (*p* = 0.046; Mann–Whitney U test) (Figure 6; Table S3).

The factorial ANOVA showed a significant decrease in CC difference (%) from preoperative data to the 1-year follow-up in both groups (F (3272) = 4.3, *p* < 0.001). The effect of “groups” was significant (F (1272) = 8.9, *p* = 0.032), with data in group N showing significantly higher CC differences than those found in group T. However, the increase in CC differences from the first post surgery measurement to the the 1-year follow-up was similar in both groups and non-significant (F (3, 272) = 0.239, *p* = 0.869) (Figure 6).

### 3.4. OL and OA Measurements

There were no statistically significant differences between groups in OL or OA at one year (*p* = 0.193 for OL and *p* = 0.066 for OA) or at any other time point (Mann–Whitney U test). Radiological results in absolute and relative values are available in Table S3.

**Figure 7:**
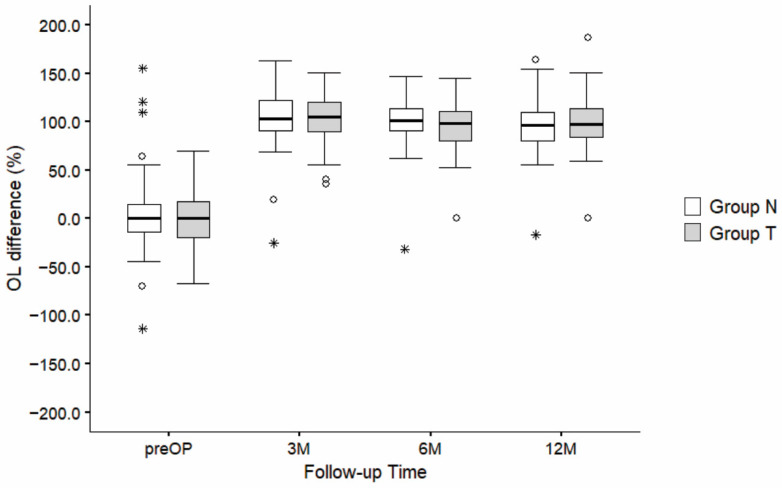
Comparison of relative OL difference (%) between group N and group T at different follow-up times (before surgery, 3 M, 6 M, and 12 M after surgery). Statistically significant differences were identified using the post-hoc Tukey test (*p* < 0.001). The boxplots represent the distribution of OL difference (%) for groups N and T, with horizontal lines showing the median values and whiskers representing 1.5 times the interquartile range (IQR). Outliers are displayed as circles (o), while asterisks (*) indicate extreme values. M—months.

Relative OL difference (%) presented a significant increase in OL from before surgery to 12 months after surgery (F (3,260) = 128.3, *p* < 0.001). However, there were no statistically significant differences between groups (F (1,260) = 0.280, *p* = 0.597) or in the interaction effects of “follow-up time and groups” (F (3,260) = 0.59, *p* = 0.619) (Figure 7).

**Figure 8:**
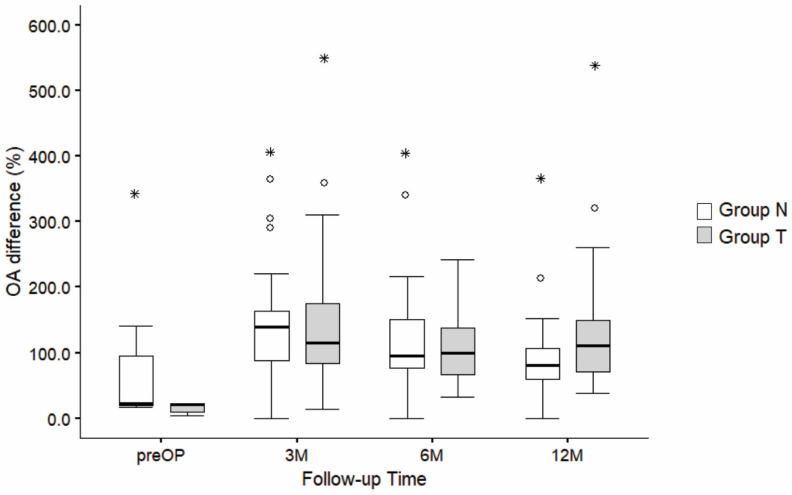
Comparison of relative OA differences (%) between group N and group T across follow-up time (before surgery, 3 M, 6 M, and 12 M after surgery). The boxplots represent the distribution of OA difference (in percentages) for groups N and T, with horizontal lines showing the median values and whiskers representing 1.5 times the interquartile range (IQR). Outliers are displayed as circles (o), while asterisks (*) indicate extreme values. Most of the preoperative data are not available because OA cannot be measured due to AC dislocation [29]. M—months.

Similar results were observed with respect to relative OA values (%), where a significant increase in OA was found over time in both groups (F (3,202) = 6.389, *p* < 0.001), again with no significance in the effect of “groups” (F (1,202) = 0.593, *p* = 0.442) or in the interaction effects of “follow-up time and groups” (F (3,202) = 2.166, *p* = 0.093) (Figure 8).

### 3.5. Complications

There was a 14.29% complication rate overall. Six out of 35 patients in group N (17.1%) and four out of 35 patients in group T (11.4%) had complications during follow-up, with no statistically significant difference between the groups (Chi-test, *p* = 0.495). Two patients in group N (5.7%) and two patients in group T (5.7%) had material-related irritation. All four underwent the removal of the superior knots after the follow-up. One patient in group N (female, 2.9%) had acute adhesive capsulitis symptoms, which resolved during 3 months of physiotherapy. One patient in group N (2.9%) had a wound infection and dehiscence that healed by secondary intention with iodine mesh and sterile dressings. Considerable loss of reduction of more than 100% was observed in seven (10%) patients at the 1-year follow-up (three patients out of 35 in group N (8.6%) and four patients out of 35 in group T (11.4%)). This was not statistically significant between groups (Chi-test, *p* = 0.69). All seven patients had a good to excellent outcome at the 1-year follow-up, with a Constant score averaging 87 points (range from 69.4 to 100) and SACS averaging 17.1 points (range from 2 to 36). Scapular dyskinesis was not appreciated in these patients. None required revision surgery.

## 4. Discussion

Options for treatment of high-grade AC joint dislocations are numerous, and there seems to be a lack of gold standard for optimal stabilization. Anatomy-mimicking procedures are now focused on repairing all three scapuloclavicular construct-stabilizing ligaments (namely the conoid, trapezoid, and AC ligament) [19,34] to avoid “left-over” horizontal instability and to maintain the clavicle’s most important strut function [38]. Yet, reducing iatrogenic trauma and minimizing the risk of complications by limiting surgical footprint has become equally important, especially in acute cases where the biological ability of native ligaments to heal is important to consider.

Our results show that there is no clear advantage in AC augmentation to the scapuloclavicular stabilization construct regarding clinical results or radiological measurements for horizontal stability. However, even with a similar gradual loss of reduction over time in both groups, there was still better retention of CC distances at 6 months and one year for group T. There was also a tendency toward superior horizontal stability in clinical testing for the augmented group.

Pain in a reduced and stable AC joint after surgery is a poorly understood inconvenience that might greatly affect short- and long-term outcomes. It has been our concern in clinical practice that additional iatrogenic trauma and overtightening of the AC joint might contribute to pain directly over the AC joint as well as to shoulder pain in general. With this notion considered, it was not a surprise that pain level throughout follow-up was higher in group T but was overall not significant in comparison to group N. In a retrospective study by Maziak et al., where factors predicting outcome in Rockwood grade V were sought, CC stabilization without AC augmentation was associated with more DPT (*p* = 0.039), and pain was encountered more commonly in patients with DPT (p_TS_ = 0.049; p_ACJI_ re 0.038; p_TS_ represents the *p*-value calculated from a subsection of the Taft score (TS); p_ACJI_ represents the *p*-value calculated from a subsection of the ACJI score) [21]. Although not directly comparable, this would suggest that there is less DPT and, subsequently, less pain in cases with a more stable AC joint where AC augmentation is used. This is opposite to our findings; however, overtightening of the joint and iatrogenic trauma may very well be surgical technique dependent.

Cisneros et al. wanted to know how much DPT remains after surgical AC stabilization. They compared four types of fixation techniques—acute single CC fixation, acute double CC fixation, acute fixation with an AC hook plate, and chronic fixation with single CC fixation and tendon graft. One-fifth of patients (18.87%) had developed DPT and were associated with worse results in the DASH score (*p* = 0.049). Among these, double CC stabilization had the least DPT. Other clinical scores, however, did not show a statistically significant correlation between the final result and DPT, as assessed on Alexander and axillary X-rays. They conclude that AC augmentation has to be considered to avoid negative impact on shoulder disabilities [22].

There is a quest to find a standardized and reproducible method for quantifying DPT. Multiple radiological methods have been developed [27,28,29,30]. We decided to use a method developed by Minkus et al., where OL and OA measurements for DPT were found to have a moderate-to-strong correlation with patient-reported outcomes, especially with AC joint-specific scores [29]. Values for OA in the present study differ from OL values at one year more than expected, especially in group N. We believe this could be partly explained by errors in acquiring correct projections as well as minor inaccuracies in measurement. Precise, automatic computer measurements could, at least in part, alleviate these problems.

Several articles in the literature suggest that double CC stabilization is a biomechanically strong anatomical replication of the torn CC ligaments. Theopold demonstrated the biomechanical superiority of the double CC tunnel technique with or without AC cerclage, compared to a single CC tunnel technique with AC cerclage [20]. There seemed to be no significant benefit in AC augmentation, at least when double CC fixation was used. Dyrna emphasized the importance of the synergistic effect of CC ligaments and the AC joint capsule for biomechanical stability [26]. They found that cutting the entire AC capsule reduced resistance force in translation to less than 25%, and resistance in rotation to less than 10%, compared to the native state. Reconstruction of both CC and AC ligaments was, therefore, recommended. Saier found that only the reconstruction of both CC and AC ligaments replicated a similar amplitude (10.8 mm, compared to 10.6 mm of native state), while also producing a two-fold increase in amplitude after loading compared to the native state (3.0 mm vs. 1.5 mm, respectively) [31]. Sumanont’s finite element study showed how much force is actually absorbed by the AC cerclage to protect the CC system [33]. A model with additional AC cerclage reduced the peak stress on the CC suture button complex by as much as 90%, while also reducing deformation and displacement of the AC joint.

Maziak et al. found that patients with anatomic reduction of the AC joint were 3.1 times more likely to develop DPT than patients with overreduction (*p* = 0.017) [21]. This study informed our decision to include overreduction in our clinical practice. Our method for overreduction is purely visual (by aligning the superior clavicular cortex to the superior cortex of the acromion intraoperatively), without intraoperative imaging. This method is less precise and fairly subjective, although we believe it is effective in most cases.

Voss et al. retrospectively compared patients with and without AC augmentation [39]. A single CC suspensory system and a PDS suture for AC cerclage were used. At the 2-year follow-up, the CC distance remained statistically non-significant between groups (*p* = 0.514). However, there was a statistically significant difference between groups in AC distance (*p* = 0.039). This distance increased similarly in both groups over time and was not significant after testing with multifactorial ANOVA (*p* = 0.545). This implies that primary reduction of the AC joint was better in groups with AC cerclage.

Several surgical techniques in the literature report good outcomes after AC augmentation [17,19,34,40,41], but, to our knowledge, there are no prospective studies to date comparing AC-augmented and non-augmented cohorts. Similarly to Voss’s study [39], the present study concludes that there is no clear clinical benefit from AC augmentation, despite the biomechanical evidence mentioned above.

AC stabilization surgeries are associated with a high failure rate (20.8%) and complication rate (14.2%), as well as a notable revision surgery rate (9.5%) [42]. Non-modifiable risk factors such as age and time from injury to surgery appear to be important [43] and were favorable in our research (average 42 year of age, 11.4 days from injury to surgery). Good healing potential likely contributes to the similar clinical results observed in both groups. Despite the additional iatrogenic trauma associated with AC fixation (bone tunnels, soft-tissue damage, additional artificial material), no significant difference in complication rates was observed between the augmented (11.43%) and the non-augmented groups (17.14%) in our series. There were no iatrogenic fractures encountered through follow-up due to additional bone tunnels in the AC-augmented group. Hardware and knot irritation under the skin is a well-known side effect, and quite common in practice, but knotless implants are now a promising solution to avoid material irritation and second surgery [41]. Considerable loss of reduction of more than 100% in CC distance was observed in seven (10%) patients. Five out of seven patients (71.4%) had a Constant score of more than 85 points, and none of them required revision surgery. In his series, Shin observed no statistically significant difference in Constant score (*p* = 0.17) among six (33.5%) patients who had loss of reduction (four patients (22%) between 50% and 100%, and two patients (11%) with more than 100% increase in CC distance) compared to 12 (67%) patients without loss of reduction [44]. In a study by Clavert [45], patients with complications had a lower Constant score (mean of 71 point) than those without complications (93 points). The overall complication rate was 22.4%, and clinical failure was defined as a Constant score below 85 points. Radiological failure, defined as a 50% increase in CC distance compared to the contralateral side, was found in 48 patients (41.3%). In their study, loss of reduction had a significant negative effect on Constant score (*p* = 0.024) and DASH results (*p* = 0.044).

Limitations of our study include a relatively small sample of patients with a short follow-up period (1 year after surgery), the inclusion of different degrees of dislocations, missing clinical and radiological data at some intermediate time points, and inaccuracies in acquiring precise projections, especially for OL and OA measurements. While the power calculation was based on primary clinical outcomes, it may have been underpowered to detect subtle differences in radiological measurements. The clinical significance of the slightly lower CC distance in group T remains questionable. We also acknowledge the subjectivity of the drawer test for residual horizontal instability, and believe more precise, standardized clinical tests with quantifiable outcomes should be sought.

## 5. Conclusions

We conclude that the final clinical outcome is comparable, wheatear AC augmentation is added to the double CC suspensory construct or not. However, patients in the augmented group reported higher levels of pain throughout the follow-up period. On the other hand, there was a tendency toward better clinical horizontal stability in this group, albeit without statistical significance. Radiologically, we found no clear evidence that AC augmentation improves horizontal stability. Nevertheless, there was a statistically significant better retention of CC distance in group T toward the end of the follow-up period. Therefore, the use of AC augmentation could be recommended.

## Figures and Tables

**Table 1:** Inclusion and exclusion criteria.

Inclusion Criteria	Exclusion Criteria
Acute AC joint dislocation (<3 weeks after injury)	Chronic injury (>3 weeks after injury)
Modified Rockwood classification IIIb, IV, V, or VI	Modified Rockwood classification I, II, or IIIa
Patient aged 18 to 65 years	Immature bone (open growth plates)
Compliance with verbal and written instructions	Open AC joint dislocation Gross damage or infection of skin in the operative area
Normal shoulder function without chronic pain prior to injury	Previous injuries or surgeries in the shoulder area that would adversely influence rehabilitation outcome

**Table 2:** Demographic data analysis for group N and group T.

Characteristic	Group N	Group T	*p*-Value
Gender (male:female)	34:1	35:0	/
Age (mean ± SD; years)	42 ± 11	42 ± 11	0.889
BMI (mean ± SD; kg/m^2^)	27 ± 3	27 ± 3	0.680
Time from injury to surgery (mean ± SD; days)	10 ± 5	12 ± 6	0.381
Side of injury (right:left; *n* (%))	19 (27.1): 16 (22.9)	20 (28.6): 15 (21.4)	0.500
Rockwood classification (IIIb:V; *n* (%))	9 (12.9): 26 (37.1)	12 (17.1): 23 (32.9)	0.603
Incidence of comorbidities (*n* (%))	6 (8.6)	2 (2.9)	0.133
Smoking (*n* (%))	1 (1.4)	1 (1.4)	0.754

SD—standard deviation; n = number of patients; N = no-augmentation group, T = tape-augmentation group, BMI—body mass index.

**Table 3:** Analysis results for primary clinical outcome measures (Constant and SACS) at 3, 6, and 12 months postoperatively.

Characteristic	Group N Mean ± SD	Group T Mean ± SD	*p*-Value
Constant (3 M)	66 ± 18	62 ± 16	0.251
Constant (6 M)	83 ± 11	81 ± 10	0.309
Constant (12 M)	89 ± 9	89 ± 10	0.664
SACS (3 M)	36 ± 22	36 ± 18	0.787
SACS (6 M)	23 ± 16	24± 18	0.745
SACS (12 M)	14 ± 10	16 ± 13	0.518

SD—standard deviation; M = months; N = no-augmentation group, T = tape-augmentation group. Mann–Whitney U test.

**Table 4:** Residual horizontal instability clinical testing analysis at the 1-year follow-up. Stable: no movement in clinical testing; partially stable: posterior dislocation less than one clavicle’s width; completely unstable: posterior dislocation more than one clavicle’s width.

Clinical Horizontal Stability Assessment	Group N	Group T	*p*-Value
Stable (*n* (%))	19 (27.9%)	28 (41.2%)	0.061
Partially unstable (*n* (%))	12 (17.6%)	5 (7.4%)	
Completely unstable (*n* (%))	3 (4.4%)	1 (1.5%)	

n = number of patients; N = no-augmentation group, T = tape-augmentation group. Chi-square test.

## Data Availability

The original contributions presented in this study are included in the article/Supplementary Materials. Further inquiries can be directed to the corresponding author.

## Supplementary Materials

The following supporting information can be downloaded at: https://www.mdpi.com/article/10.3390/jcm14093161/s1, Figure S1: Comparison of CC difference (mm) between Group N and Group T across follow-up time points (before surgery, 3 M, 6 M, and 12 M after surgery). The boxplots represent the distribution of CC difference in mm for Groups N and T, with horizontal lines showing the median values and whiskers representing 1.5 times the interquartile range (IQR). Statistically significant results are displayed above 6M and 12M as *p*-values. Outliers are displayed as circles (o), and asterisks (*) indicate extreme values. M—months; Table S1: Surgery data; Table S2: Test results of PROMs, pain level and strength (at 1-year). Significant differences are noted in red; Table S3: Radiological measurements of CC, OA and OL for group N and group T.
