# Supplementary material for: No Short-Term Effects of Acromioclavicular Joint Augmentation in Acute Acromioclavicular Joint Stabilization Surgery: A Randomized Controlled Clinical Trial on 70 Patients
Source: J Clin Med. 2025 May 2;14(9):3161. doi: 10.3390/jcm14093161 (PMC12072358; doi:10.3390/jcm14093161)
Supplement: Supplementary file 1 [file jcm-14-03161-s001.zip › jcm-3585875-supplementary.pdf]

Supplementary Materials

Surgical technique description.

Patients were positioned in a beach chair position. Approximately 6 - 8 cm strap-line incision above lateral clavicle was made. Subcutaneous tissue was dissected to muscle fascia, which was then incised longitudinally over the superior clavicle. AC joint was fully visualised and intraarticular disc, if degenerated, was extirpated. Vertical bone tunnels were made on each side of the AC joint in group T. Shuttle sutures were passed through bone tunnels. No tunnels were drilled for group N. Clavicle was anatomically reduced and temporarily fixated with a K-wire through acromion. Marks for bone tunnels were made 2.5 and 4.5 cm medially from the lateral clavicle edge (1). The second part was arthroscopic. A standard posterior viewing portal and anterior working portal were established. A diagnostic arthroscopy was done, describing any intraarticular pathology. Coracoid process was then located. At this point the 30º arthroscope was repositioned to the anterolateral portal and coracoid under surface was debrided to its base. A 90º drill guide with 2.4 mm cannulated drills was used for bone tunnels from marked points on clavicle aimed towards the base of coracoid. A nitinol suture passing wire was inserted in the cannulated drill and retrieved through the anterior portal. The cannulated drill was removed. A strong shuttle suture was attached to the wire and pulled upwards through the bone tunnel (implant position is displayed in figure 2 and 3). A second button was then added from the superior side and several knots were tied to secure the construct. Medial suspensory system was done first after which the process was replicated for the lateral one. K-wire securing reduction was removed prior to knot tying of both systems and manual reposition with intentional minor over reduction (2) was obtained with direct visualisation (no image intensifier utilised).

The last part of the surgery was done differently, depending on the allocated group. This was the end of surgery for group N. In group T a FiberTape® was shuttled through bone tunnels on each side of the AC joint and tied on the posterosuperior side of the joint (*Figure 2 and 3b*).

Deltotrapezoid fascia was closed with interrupted double-looped absorbable sutures. In the lateral part the superior AC ligament was incorporated in the fascia suture and was as such repaired in the sutured construct. All incisions were closed by layers in a standard fashion.

Supplementary Table S1. Surgery data.

	Group N					Group T					p-value
	**Mean**	**SD**	**Median**	**Q1**	**Q3**	**Mean**	**SD**	**Median**	**Q1**	**Q3**	
Incision length (cm)	8.3	1.6	9.0	8.0	9.0	8.7	1.2	9.0	8.5	9.5	0,422
Surgical time (min)	76	18	75	60	90	81	13	80	70	90	0,125

SD – standard deviation; N – no-augmentation group (Group N); T – tape-augmentation group (Group T); BMI – body mass index. p-values are calculated using the Mann–Whitney U test.

Supplementary Table S2. Test results of PROMs, pain level and strength (at 1-year). Significant difference are noted in red.

		Group N							Group T							
**Measurment**	**FU**	**Mean**	**SD**	**Min**	**Max**	**Median**	**Q1**	**Q3**	**Mean**	**SD**	**Min**	**Max**	**Median**	**Q1**	**Q3**	***p*-Value**
	3M	7	3	1	12	8	5	9	7	3	1	12	7	6	9	0.806
SST	6M	10	2	5	12	11	9	12	10	2	5	12	10	8	12	0.411
	12M	11	1	8	12	11	10	12	11	2	4	12	11	11	12	0.789
SSV	3M	68	23	10	95	70	60	90	65	19	15	90	70	50	80	0.237

	6M	84	11	55	100	90	80	90	82	13	35	98	85	75	90	0.480
	12M	91	7	70	100	90	90	95	89	11	40	100	90	85	95	0.607
DASH	3M	30	21	0	74	24	14	49	34	21	0	83	31	14	50	0.267
	6M	14	17	0	81	8	3	21	18	16	0	68	13	7	32	0.093
	12M	10	16	0	66	4	1	13	9	12	0	53	7	1	13	0.443
ACJI	3M	61	14	35	90	65	52	69	62	15	44	95	57	50	65	0.831
	6M	74	15	49	100	74	65	85	72	18	44	100	70	58	85	0.599
	12M	74	20	25	100	75	64	88	80	15	51	100	82	68	94	0.346
Pain level	3M	4	3	0	12	5	2	6	4	3	0	14	3	2	6	0.990
	6M	2	2	0	6	1	0	3	3	2	0	9	2	2	5	**0.046**
	12M	2	2	0	5	1	0	2	2	3	0	12	1	0	2	0.970
Stregth at 1 year (%)		92	15	59	121	95	80	101	87	17	29	116	91	81	99	0.257

SST – Simple Shoulder Test; SSV – Subjective Shoulder Value; DASH – Disabilities of the Arm, Shoulder and Hand score; ACJI – Acromioclavicular Joint Instability score; ACJ – Acromioclavicular Joint (clinical assessment); SD – Standard Deviation; Q1/Q3 – First/Third Quartile; FU – Follow-up Time; Group N – No-augmentation group; Group T – Tape-augmentation group. Strength is expressed as a percentage of the contralateral (non-operated) side. p-values were calculated using the Mann–Whitney U test.

Supplementary Table S3. Radiological measurements of CC, OA and OL for group N and group T. Significant difference are noted in red.

Measurment	FU	Group N Mean	SD	Median	Q1	Q3	Group T Mean	SD	Median	Q1	Q3	*p*-Value
CC difference (mm)	preOP	10.2	3.7	9.5	7.3	12.8	9.2	2.6	9.1	7.3	10.8	0.445
	3M	2.1	1.8	1.5	0.8	2.8	1.8	1.6	1.4	0.5	3.2	0.233
	6M	3.1	2.4	2.8	1.4	4.6	1.8	1.6	1.6	0.5	2.7	**0.027**
	12M	3.8	3.0	2.9	1.5	4.7	2.4	2.1	1.9	1.0	2.9	**0.043**
CC difference (%)	preOP	233.9	76.4	213.2	177.8	289.9	220.2	69.4	205.1	179.8	232.9	0.597
	3M	117.3	34.8	109.8	93.9	131.8	103.1	38.2	101.4	79.7	118.1	0.231
	6M	141.0	52.1	122.7	111.3	159.1	115.9	31.5	109.1	97.1	131.7	**0.047**
	12M	155.0	69.0	141.9	118.3	163.5	130.1	46.5	118.7	101.8	141.7	**0.046**
OL difference (mm)	preOP	10.9	4.1	11.6	7.9	13.6	11.1	3.9	11.5	7.9	13.2	0.583
	3M	2.2	3.0	1.7	0.8	2.9	2.1	1.9	1.5	0.6	3.0	0.956
	6M	1.6	1.8	1.3	0.8	2.3	2.2	2.6	1.6	0.8	2.6	0.315
	12M	2.5	2.5	1.8	0.8	3.3	2.6	2.6	1.9	0.8	3.5	0.722
OL difference (%)	preOP	7.2	50.2	0.0	-14.0	21.4	-1.4	33.8	0.0	-19.8	17.7	0.963
	3M	100.5	35.7	103.2	90.5	122.0	103.7	27.6	104.7	89.3	120.3	0.411
	6M	99.6	30.6	101.6	90.7	115.1	92.3	32.0	98.7	80.2	110.6	0.355
	12M	96.4	33.2	96.4	79.4	111.2	100.1	31.4	97.7	82.6	114.0	0.193
OA difference (mm^2)	preOP	51.4	29.5	39.0	36.8	87.1	88.1	35.5	96.4	60.8	118.3	0.060
	3M	41.6	32.9	34.4	17.9	56.3	39.9	30.6	33.2	16.1	57.2	0.979
	6M	36.6	32.6	25.1	13.9	52.9	35.5	29.0	24.6	16.3	52.7	0.814
	12M	37.2	42.7	20.6	6.6	52.7	37.3	27.3	34.3	15.7	48.2	0.455
OA difference (%)	preOP	87.5	121.0	21.9	17.7	141.0	15.5	8.0	21.2	7.8	21.9	0.220
	3M	149.9	90.7	138.8	87.7	163.6	151.8	108.9	115.5	83.4	174.4	0.882
	6M	119.1	83.1	95.1	76.3	151.7	104.6	50.7	99.8	67.0	140.6	0.756
	12M	90.6	65.7	80.8	59.4	106.6	130.3	96.7	110.4	71.0	149.5	0.066

SD – Standard Deviation; Q1/Q3 – First/Third Quartile; FU – Follow-up; N – No-augmentation group; T – Tape-augmentation group; CC – Coracoclavicular; OL – Over-reduction Length; OA – Over-reduction Area. Measurements are expressed in millimeters (mm), square millimeters (mm^2), or percentages (%), as appropriate. Time points: preOP – Preoperative; 3M – 3 Months; 6M – 6 Months; 12M – 12 Months after surgery. p-values were calculated using the Mann–Whitney U test.

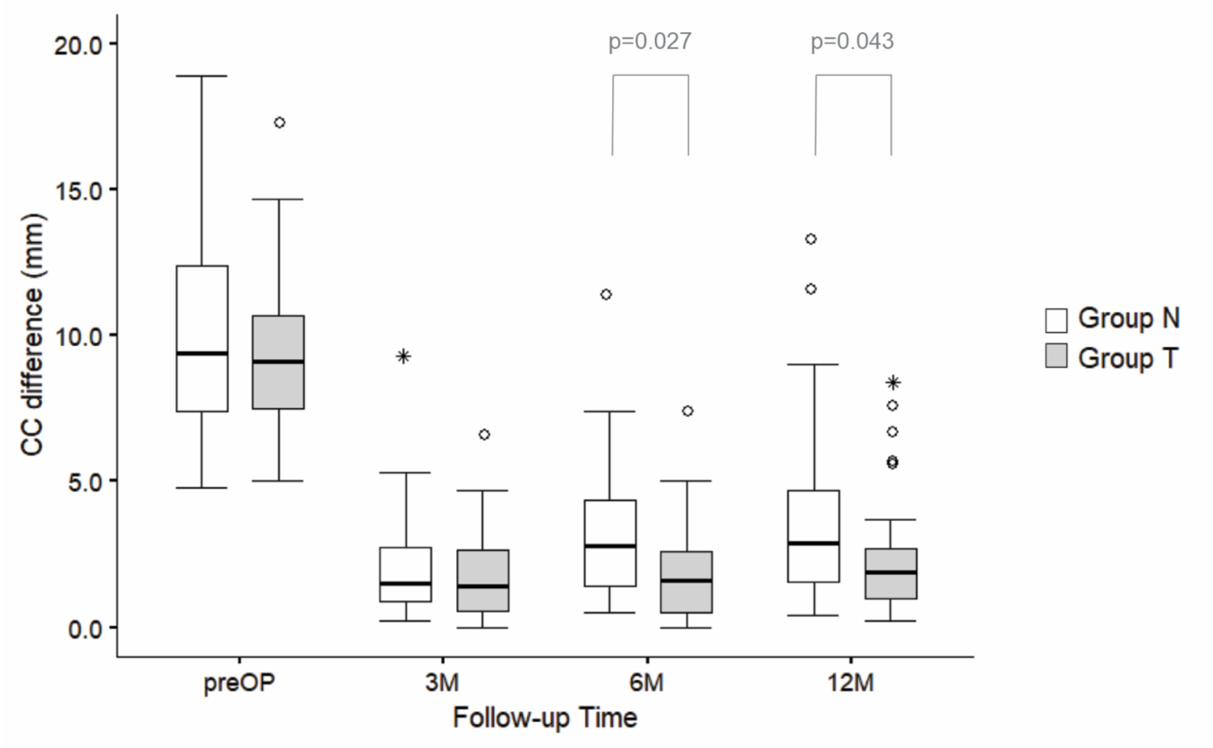

Supplementary Figure S1. Comparison of CC difference (mm) between Group N and Group T across follow-up time points (before surgery. 3 M. 6 M. and 12 M after surgery). The boxplots represent the distribution of CC difference in mm for Groups N and T. with horizontal lines showing the median values and whiskers representing 1.5 times the interquartile range (IQR). Statistically significant results are displayed above 6M and 12M as p-values. Outliers are displayed as circles (o). and asterisks (*) indicate extreme values. M—months.
